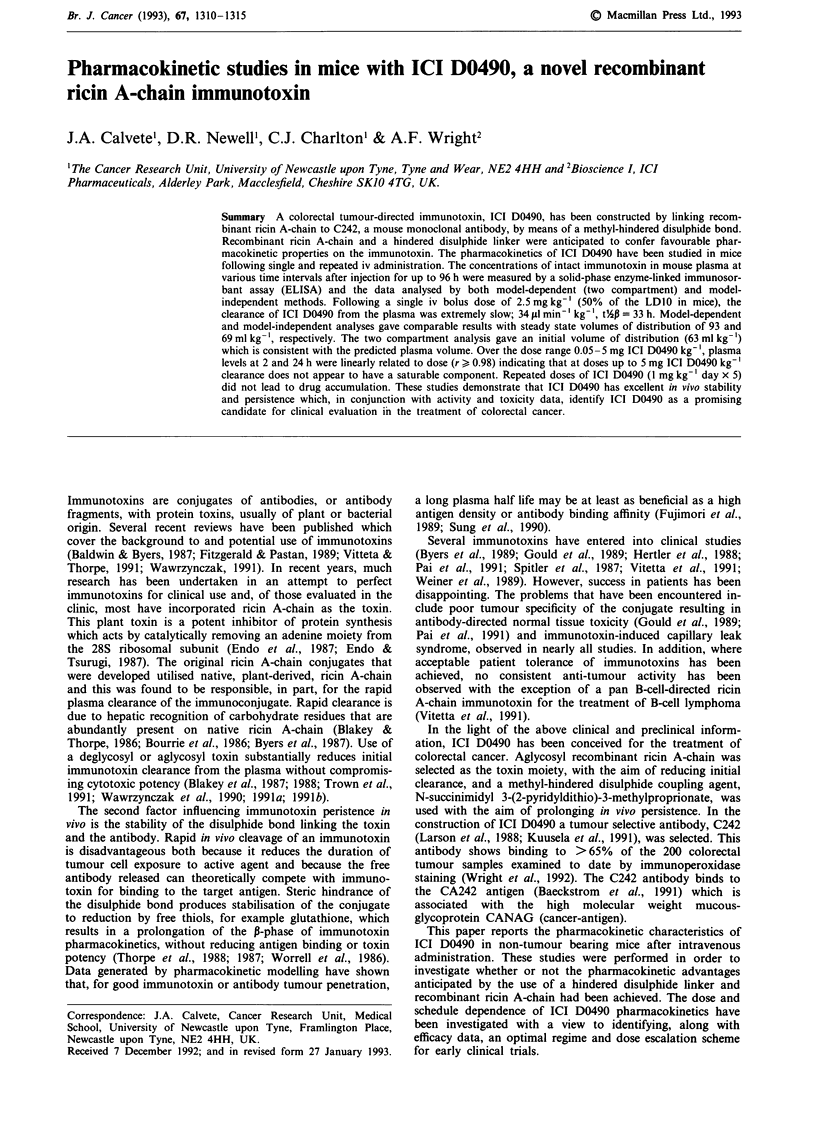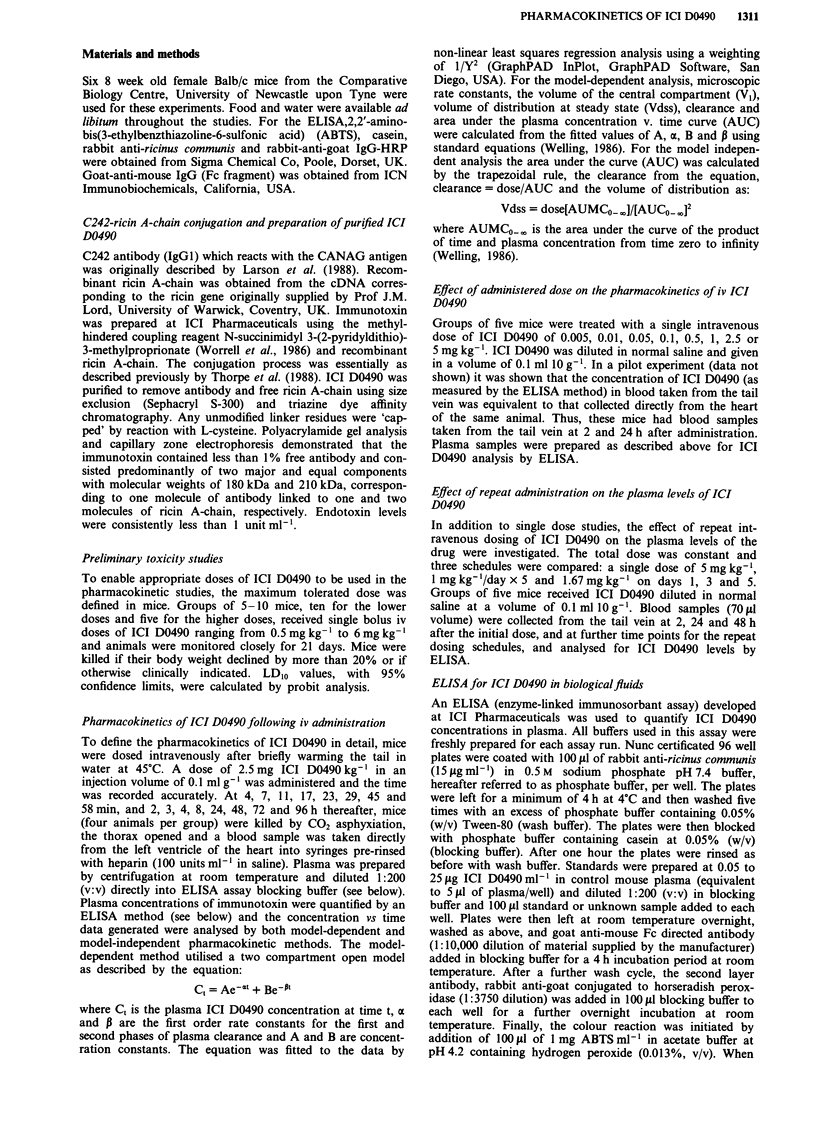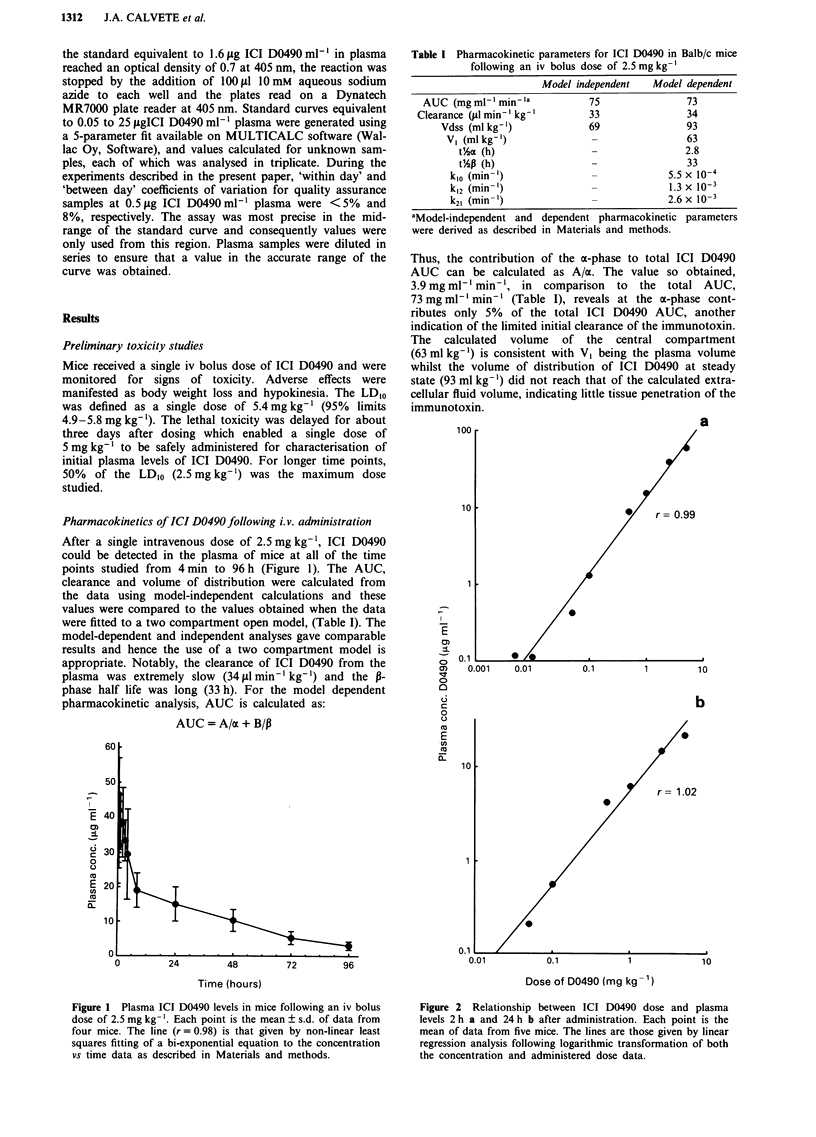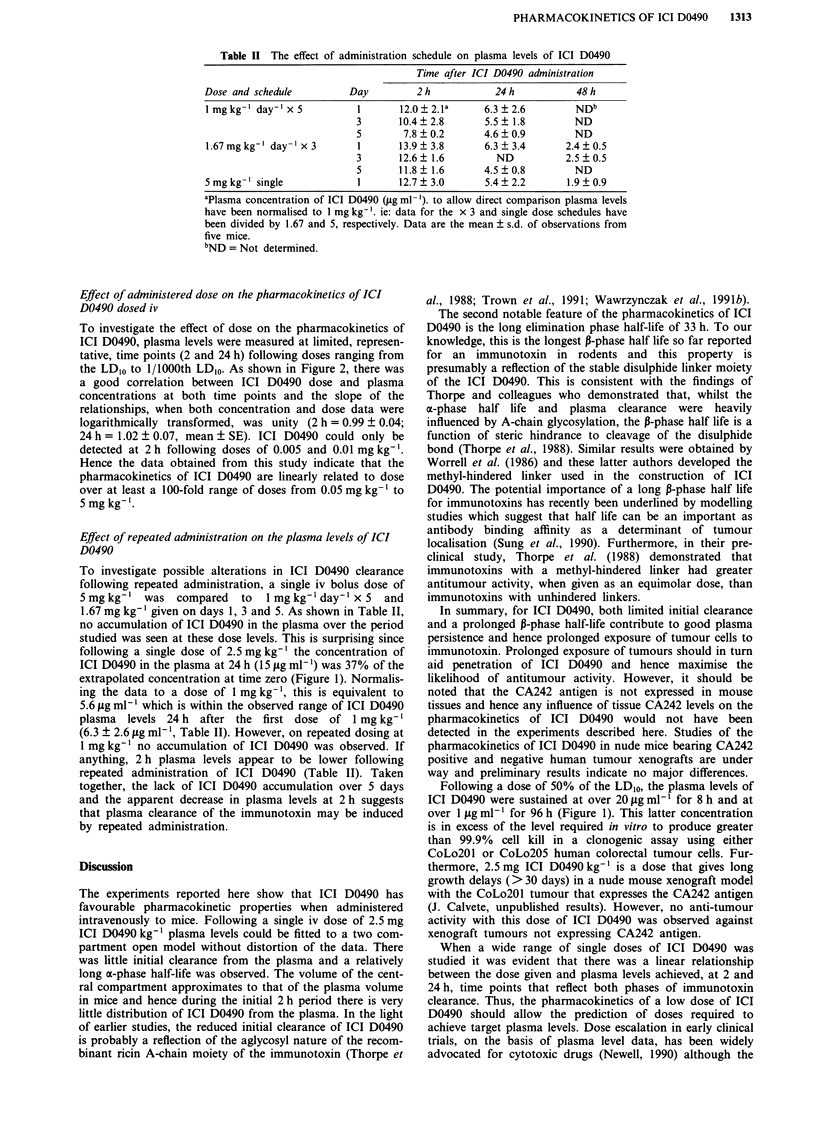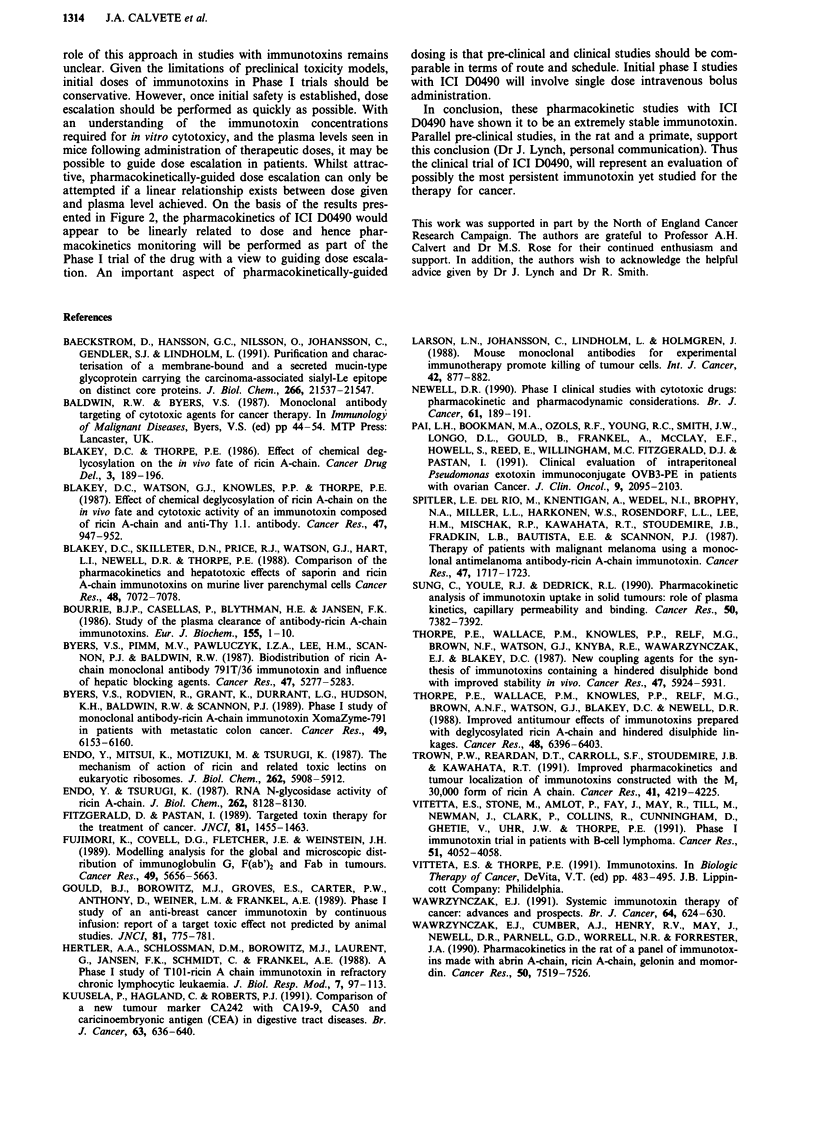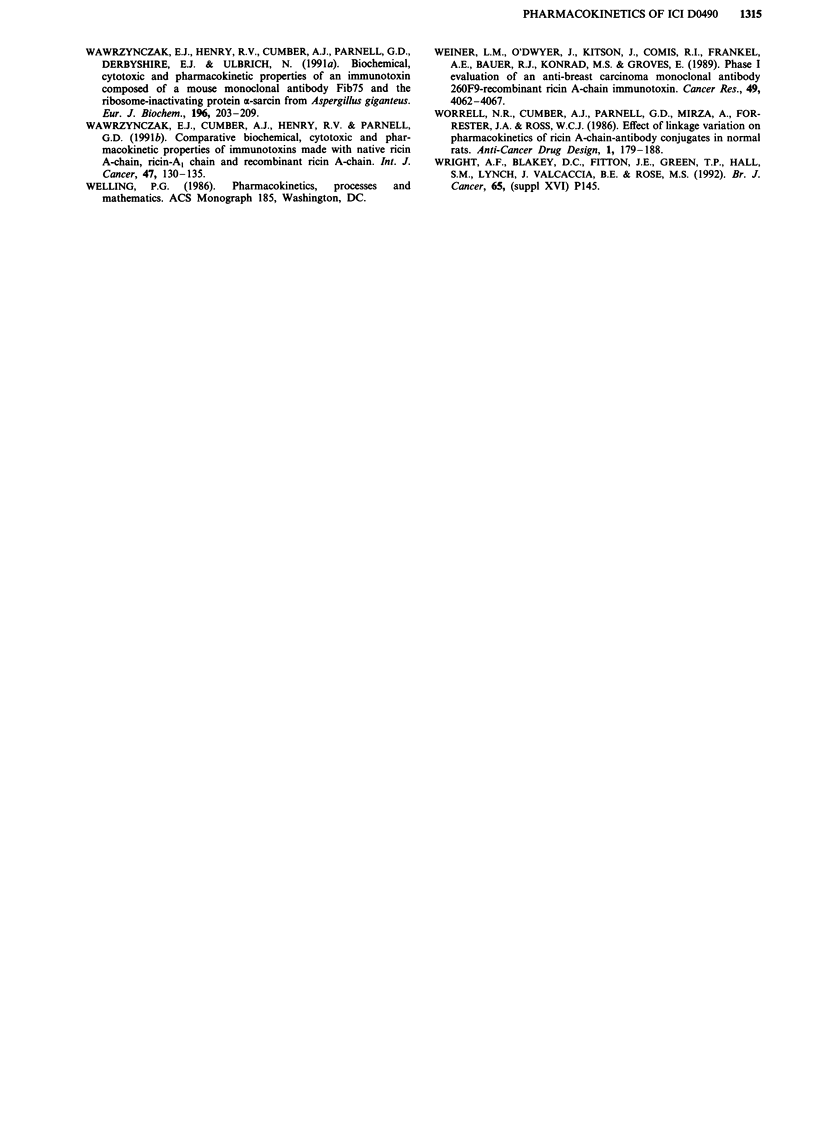# Pharmacokinetic studies in mice with ICI D0490, a novel recombinant ricin A-chain immunotoxin.

**DOI:** 10.1038/bjc.1993.243

**Published:** 1993-06

**Authors:** J. A. Calvete, D. R. Newell, C. J. Charlton, A. F. Wright

**Affiliations:** Cancer Research Unit, University of Newcastle upon Tyne, Tyne and Wear, UK.

## Abstract

A colorectal tumour-directed immunotoxin, ICI D0490, has been constructed by linking recombinant ricin A-chain to C242, a mouse monoclonal antibody, by means of a methyl-hindered disulphide bond. Recombinant ricin A-chain and a hindered disulphide linker were anticipated to confer favourable pharmacokinetic properties on the immunotoxin. The pharmacokinetics of ICI D0490 have been studied in mice following single and repeated i.v. administration. The concentrations of intact immunotoxin in mouse plasma at various time intervals after injection for up to 96 h were measured by a solid-phase enzyme-linked immunosorbent assay (ELISA) and the data analysed by both model-dependent (two compartment) and model-independent methods. Following a single i.v. bolus dose of 2.5 mg kg-1 (50% of the LD10 in mice), the clearance of ICI D0490 from the plasma was extremely slow; 34 microliters min-1 kg-1, t1/2 beta = 33 h. Model-dependent and model-independent analyses gave comparable results with steady state volumes of distribution of 93 and 69 ml kg-1, respectively. The two compartment analysis gave an initial volume of distribution (63 ml kg-1) which is consistent with the predicted plasma volume. Over the dose range 0.05-5 mg ICI D0490 kg-1, plasma levels at 2 and 24 h were linearly related to dose (r > or = 0.98) indicating that at doses up to 5 mg ICI D0490 kg-1 clearance does not appear to have a saturable component. Repeated doses of ICI D0490 (1 mg kg-1 day x 5) did not lead to drug accumulation. These studies demonstrate that ICI D0490 has excellent in vivo stability and persistence which, in conjunction with activity and toxicity data, identify ICI D0490 as a promising candidate for clinical evaluation in the treatment of colorectal cancer.


Br. J. Cancer (1993), 67, 1310-1315 © Macmillan Press Ltd., 1993

### Pharmacokinetic studies in mice with ICI D0490, a novel recombinant ricin A-chain immunotoxin

J.A. Calvete[1], D.R. Newell[1], C.J. Charlton[1] & A.F. Wright[2]

[1]*The Cancer Research Unit, University of Newcastle upon Tyne, Tyne and Wear, NE2 4HH and* [2]*Bioscience I, ICI Pharmaceuticals, Alderley Park, Macclesfield, Cheshire SK10 4TG, UK.*

**Summary** A colorectal tumour-directed immunotoxin, ICI D0490, has been constructed by linking recombinant ricin A-chain to C242, a mouse monoclonal antibody, by means of a methyl-hindered disulphide bond. Recombinant ricin A-chain and a hindered disulphide linker were anticipated to confer favourable pharmacokinetic properties on the immunotoxin. The pharmacokinetics of ICI D0490 have been studied in mice following single and repeated iv administration. The concentrations of intact immunotoxin in mouse plasma at various time intervals after injection for up to 96 h were measured by a solid-phase enzyme-linked immunosorbant assay (ELISA) and the data analysed by both model-dependent (two compartment) and model-independent methods. Following a single iv bolus dose of 2.5 mg kg$^{-1}$ (50% of the LD10 in mice), the clearance of ICI D0490 from the plasma was extremely slow; 34 $\mu$l min$^{-1}$ kg$^{-1}$, t½$\beta$ = 33 h. Model-dependent and model-independent analyses gave comparable results with steady state volumes of distribution of 93 and 69 ml kg$^{-1}$, respectively. The two compartment analysis gave an initial volume of distribution (63 ml kg$^{-1}$) which is consistent with the predicted plasma volume. Over the dose range 0.05-5 mg ICI D0490 kg$^{-1}$, plasma levels at 2 and 24 h were linearly related to dose ($r \geqslant 0.98$) indicating that at doses up to 5 mg ICI D0490 kg$^{-1}$ clearance does not appear to have a saturable component. Repeated doses of ICI D0490 (1 mg kg$^{-1}$ day × 5) did not lead to drug accumulation. These studies demonstrate that ICI D0490 has excellent *in vivo* stability and persistence which, in conjunction with activity and toxicity data, identify ICI D0490 as a promising candidate for clinical evaluation in the treatment of colorectal cancer.

Immunotoxins are conjugates of antibodies, or antibody fragments, with protein toxins, usually of plant or bacterial origin. Several recent reviews have been published which cover the background to and potential use of immunotoxins (Baldwin & Byers, 1987; Fitzgerald & Pastan, 1989; Vitteta & Thorpe, 1991; Wawrzynczak, 1991). In recent years, much research has been undertaken in an attempt to perfect immunotoxins for clinical use and, of those evaluated in the clinic, most have incorporated ricin A-chain as the toxin. This plant toxin is a potent inhibitor of protein synthesis which acts by catalytically removing an adenine moiety from the 28S ribosomal subunit (Endo *et al.*, 1987; Endo & Tsurugi, 1987). The original ricin A-chain conjugates that were developed utilised native, plant-derived, ricin A-chain and this was found to be responsible, in part, for the rapid plasma clearance of the immunoconjugate. Rapid clearance is due to hepatic recognition of carbohydrate residues that are abundantly present on native ricin A-chain (Blakey & Thorpe, 1986; Bourrie *et al.*, 1986; Byers *et al.*, 1987). Use of a deglycosyl or aglycosyl toxin substantially reduces initial immunotoxin clearance from the plasma without compromising cytotoxic potency (Blakey *et al.*, 1987; 1988; Trown *et al.*, 1991; Wawrzynczak *et al.*, 1990; 1991*a*; 1991*b*).

The second factor influencing immunotoxin peristence *in vivo* is the stability of the disulphide bond linking the toxin and the antibody. Rapid *in vivo* cleavage of an immunotoxin is disadvantageous both because it reduces the duration of tumour cell exposure to active agent and because the free antibody released can theoretically compete with immunotoxin for binding to the target antigen. Steric hindrance of the disulphide bond produces stabilisation of the conjugate to reduction by free thiols, for example glutathione, which results in a prolongation of the $\beta$-phase of immunotoxin pharmacokinetics, without reducing antigen binding or toxin potency (Thorpe *et al.*, 1988; 1987; Worrell *et al.*, 1986). Data generated by pharmacokinetic modelling have shown that, for good immunotoxin or antibody tumour penetration, a long plasma half life may be at least as beneficial as a high antigen density or antibody binding affinity (Fujimori *et al.*, 1989; Sung *et al.*, 1990).

Several immunotoxins have entered into clinical studies (Byers *et al.*, 1989; Gould *et al.*, 1989; Hertler *et al.*, 1988; Pai *et al.*, 1991; Spitler *et al.*, 1987; Vitetta *et al.*, 1991; Weiner *et al.*, 1989). However, success in patients has been disappointing. The problems that have been encountered include poor tumour specificity of the conjugate resulting in antibody-directed normal tissue toxicity (Gould *et al.*, 1989; Pai *et al.*, 1991) and immunotoxin-induced capillary leak syndrome, observed in nearly all studies. In addition, where acceptable patient tolerance of immunotoxins has been achieved, no consistent anti-tumour activity has been observed with the exception of a pan B-cell-directed ricin A-chain immunotoxin for the treatment of B-cell lymphoma (Vitetta *et al.*, 1991).

In the light of the above clinical and preclinical information, ICI D0490 has been conceived for the treatment of colorectal cancer. Aglycosyl recombinant ricin A-chain was selected as the toxin moiety, with the aim of reducing initial clearance, and a methyl-hindered disulphide coupling agent, N-succinimidyl 3-(2-pyridyldithio)-3-methylproprionate, was used with the aim of prolonging *in vivo* persistence. In the construction of ICI D0490 a tumour selective antibody, C242 (Larson *et al.*, 1988; Kuusela *et al.*, 1991), was selected. This antibody shows binding to >65% of the 200 colorectal tumour samples examined to date by immunoperoxidase staining (Wright *et al.*, 1992). The C242 antibody binds to the CA242 antigen (Baeckstrom *et al.*, 1991) which is associated with the high molecular weight mucous-glycoprotein CANAG (cancer-antigen).

This paper reports the pharmacokinetic characteristics of ICI D0490 in non-tumour bearing mice after intravenous administration. These studies were performed in order to investigate whether or not the pharmacokinetic advantages anticipated by the use of a hindered disulphide linker and recombinant ricin A-chain had been achieved. The dose and schedule dependence of ICI D0490 pharmacokinetics have been investigated with a view to identifying, along with efficacy data, an optimal regime and dose escalation scheme for early clinical trials.

Correspondence: J.A. Calvete, Cancer Research Unit, Medical School, University of Newcastle upon Tyne, Framlington Place, Newcastle upon Tyne, NE2 4HH, UK.
Received 7 December 1992; and in revised form 27 January 1993.

### Materials and methods

Six 8 week old female Balb/c mice from the Comparative Biology Centre, University of Newcastle upon Tyne were used for these experiments. Food and water were available *ad libitum* throughout the studies. For the ELISA,2,2′-amino-bis(3-ethylbenzthiazoline-6-sulfonic acid) (ABTS), casein, rabbit anti-*ricinus communis* and rabbit-anti-goat IgG-HRP were obtained from Sigma Chemical Co, Poole, Dorset, UK. Goat-anti-mouse IgG (Fc fragment) was obtained from ICN Immunobiochemicals, California, USA.

### *C242-ricin A-chain conjugation and preparation of purified ICI D0490*

C242 antibody (IgG1) which reacts with the CANAG antigen was originally described by Larson *et al.* (1988). Recombinant ricin A-chain was obtained from the cDNA corresponding to the ricin gene originally supplied by Prof J.M. Lord, University of Warwick, Coventry, UK. Immunotoxin was prepared at ICI Pharmaceuticals using the methyl-hindered coupling reagent N-succinimidyl 3-(2-pyridyldithio)-3-methylproprionate (Worrell *et al.*, 1986) and recombinant ricin A-chain. The conjugation process was essentially as described previously by Thorpe *et al.* (1988). ICI D0490 was purified to remove antibody and free ricin A-chain using size exclusion (Sephacryl S-300) and triazine dye affinity chromatography. Any unmodified linker residues were 'capped' by reaction with L-cysteine. Polyacrylamide gel analysis and capillary zone electrophoresis demonstrated that the immunotoxin contained less than 1% free antibody and consisted predominantly of two major and equal components with molecular weights of 180 kDa and 210 kDa, corresponding to one molecule of antibody linked to one and two molecules of ricin A-chain, respectively. Endotoxin levels were consistently less than 1 unit $ml^{-1}$.

### *Preliminary toxicity studies*

To enable appropriate doses of ICI D0490 to be used in the pharmacokinetic studies, the maximum tolerated dose was defined in mice. Groups of 5–10 mice, ten for the lower doses and five for the higher doses, received single bolus iv doses of ICI D0490 ranging from 0.5 mg $kg^{-1}$ to 6 mg $kg^{-1}$ and animals were monitored closely for 21 days. Mice were killed if their body weight declined by more than 20% or if otherwise clinically indicated. $LD_{10}$ values, with 95% confidence limits, were calculated by probit analysis.

### *Pharmacokinetics of ICI D0490 following iv administration*

To define the pharmacokinetics of ICI D0490 in detail, mice were dosed intravenously after briefly warming the tail in water at 45°C. A dose of 2.5 mg ICI D0490 $kg^{-1}$ in an injection volume of 0.1 ml $g^{-1}$ was administered and the time was recorded accurately. At 4, 7, 11, 17, 23, 29, 45 and 58 min, and 2, 3, 4, 8, 24, 48, 72 and 96 h thereafter, mice (four animals per group) were killed by $CO_2$ asphyxiation, the thorax opened and a blood sample was taken directly from the left ventricle of the heart into syringes pre-rinsed with heparin (100 units $ml^{-1}$ in saline). Plasma was prepared by centrifugation at room temperature and diluted 1:200 (v:v) directly into ELISA assay blocking buffer (see below). Plasma concentrations of immunotoxin were quantified by an ELISA method (see below) and the concentration *vs* time data generated were analysed by both model-dependent and model-independent pharmacokinetic methods. The model-dependent method utilised a two compartment open model as described by the equation:

$$C_t = Ae^{-\alpha t} + Be^{-\beta t}$$

where $C_t$ is the plasma ICI D0490 concentration at time t, $\alpha$ and $\beta$ are the first order rate constants for the first and second phases of plasma clearance and A and B are concentration constants. The equation was fitted to the data by non-linear least squares regression analysis using a weighting of $1/Y^2$ (GraphPAD InPlot, GraphPAD Software, San Diego, USA). For the model-dependent analysis, microscopic rate constants, the volume of the central compartment ($V_1$), volume of distribution at steady state (Vdss), clearance and area under the plasma concentration v. time curve (AUC) were calculated from the fitted values of A, $\alpha$, B and $\beta$ using standard equations (Welling, 1986). For the model independent analysis the area under the curve (AUC) was calculated by the trapezoidal rule, the clearance from the equation, clearance = dose/AUC and the volume of distribution as:

$$Vdss = dose[AUMC_{0-\infty}]/[AUC_{0-\infty}]^2$$

where $AUMC_{0-\infty}$ is the area under the curve of the product of time and plasma concentration from time zero to infinity (Welling, 1986).

### *Effect of administered dose on the pharmacokinetics of iv ICI D0490*

Groups of five mice were treated with a single intravenous dose of ICI D0490 of 0.005, 0.01, 0.05, 0.1, 0.5, 1, 2.5 or 5 mg $kg^{-1}$. ICI D0490 was diluted in normal saline and given in a volume of 0.1 ml 10 $g^{-1}$. In a pilot experiment (data not shown) it was shown that the concentration of ICI D0490 (as measured by the ELISA method) in blood taken from the tail vein was equivalent to that collected directly from the heart of the same animal. Thus, these mice had blood samples taken from the tail vein at 2 and 24 h after administration. Plasma samples were prepared as described above for ICI D0490 analysis by ELISA.

### *Effect of repeat administration on the plasma levels of ICI D0490*

In addition to single dose studies, the effect of repeat intravenous dosing of ICI D0490 on the plasma levels of the drug were investigated. The total dose was constant and three schedules were compared: a single dose of 5 mg $kg^{-1}$, 1 mg $kg^{-1}$/day × 5 and 1.67 mg $kg^{-1}$ on days 1, 3 and 5. Groups of five mice received ICI D0490 diluted in normal saline at a volume of 0.1 ml 10 $g^{-1}$. Blood samples (70 µl volume) were collected from the tail vein at 2, 24 and 48 h after the initial dose, and at further time points for the repeat dosing schedules, and analysed for ICI D0490 levels by ELISA.

### *ELISA for ICI D0490 in biological fluids*

An ELISA (enzyme-linked immunosorbant assay) developed at ICI Pharmaceuticals was used to quantify ICI D0490 concentrations in plasma. All buffers used in this assay were freshly prepared for each assay run. Nunc certificated 96 well plates were coated with 100 µl of rabbit anti-*ricinus communis* (15 µg $ml^{-1}$) in 0.5 M sodium phosphate pH 7.4 buffer, hereafter referred to as phosphate buffer, per well. The plates were left for a minimum of 4 h at 4°C and then washed five times with an excess of phosphate buffer containing 0.05% (w/v) Tween-80 (wash buffer). The plates were then blocked with phosphate buffer containing casein at 0.05% (w/v) (blocking buffer). After one hour the plates were rinsed as before with wash buffer. Standards were prepared at 0.05 to 25 µg ICI D0490 $ml^{-1}$ in control mouse plasma (equivalent to 5 µl of plasma/well) and diluted 1:200 (v:v) in blocking buffer and 100 µl standard or unknown sample added to each well. Plates were then left at room temperature overnight, washed as above, and goat anti-mouse Fc directed antibody (1:10,000 dilution of material supplied by the manufacturer) added in blocking buffer for a 4 h incubation period at room temperature. After a further wash cycle, the second layer antibody, rabbit anti-goat conjugated to horseradish peroxidase (1:3750 dilution) was added in 100 µl blocking buffer to each well for a further overnight incubation at room temperature. Finally, the colour reaction was initiated by addition of 100 µl of 1 mg ABTS $ml^{-1}$ in acetate buffer at pH 4.2 containing hydrogen peroxide (0.013%, v/v). When the standard equivalent to 1.6 µg ICI D0490 $ml^{-1}$ in plasma reached an optical density of 0.7 at 405 nm, the reaction was stopped by the addition of 100 µl 10 mM aqueous sodium azide to each well and the plates read on a Dynatech MR7000 plate reader at 405 nm. Standard curves equivalent to 0.05 to 25 µgICI D0490 $ml^{-1}$ plasma were generated using a 5-parameter fit available on MULTICALC software (Wallac Oy, Software), and values calculated for unknown samples, each of which was analysed in triplicate. During the experiments described in the present paper, 'within day' and 'between day' coefficients of variation for quality assurance samples at 0.5 µg ICI D0490 $ml^{-1}$ plasma were $<5\%$ and 8%, respectively. The assay was most precise in the mid-range of the standard curve and consequently values were only used from this region. Plasma samples were diluted in series to ensure that a value in the accurate range of the curve was obtained.

### Results

### *Preliminary toxicity studies*

Mice received a single iv bolus dose of ICI D0490 and were monitored for signs of toxicity. Adverse effects were manifested as body weight loss and hypokinesia. The $LD_{10}$ was defined as a single dose of 5.4 mg $kg^{-1}$ (95% limits 4.9–5.8 mg $kg^{-1}$). The lethal toxicity was delayed for about three days after dosing which enabled a single dose of 5 mg $kg^{-1}$ to be safely administered for characterisation of initial plasma levels of ICI D0490. For longer time points, 50% of the $LD_{10}$ (2.5 mg $kg^{-1}$) was the maximum dose studied.

### *Pharmacokinetics of ICI D0490 following i.v. administration*

After a single intravenous dose of 2.5 mg $kg^{-1}$, ICI D0490 could be detected in the plasma of mice at all of the time points studied from 4 min to 96 h (Figure 1). The AUC, clearance and volume of distribution were calculated from the data using model-independent calculations and these values were compared to the values obtained when the data were fitted to a two compartment open model, (Table I). The model-dependent and independent analyses gave comparable results and hence the use of a two compartment model is appropriate. Notably, the clearance of ICI D0490 from the plasma was extremely slow (34 µl $min^{-1}$ $kg^{-1}$) and the $\beta$-phase half life was long (33 h). For the model dependent pharmacokinetic analysis, AUC is calculated as:

$$AUC = A/\alpha + B/\beta$$

**Figure 1** Plasma ICI D0490 levels in mice following an iv bolus dose of 2.5 mg $kg^{-1}$. Each point is the mean ± s.d. of data from four mice. The line ($r = 0.98$) is that given by non-linear least squares fitting of a bi-exponential equation to the concentration *vs* time data as described in Materials and methods.

**Table I** Pharmacokinetic parameters for ICI D0490 in Balb/c mice following an iv bolus dose of 2.5 mg $kg^{-1}$

| | *Model independent* | *Model dependent* |
|---|---|---|
| AUC (mg $ml^{-1}$ $min^{-1}$)[a] | 75 | 73 |
| Clearance (µl $min^{-1}$ $kg^{-1}$) | 33 | 34 |
| Vdss (ml $kg^{-1}$) | 69 | 93 |
| $V_1$ (ml $kg^{-1}$) | – | 63 |
| $t\frac{1}{2}\alpha$ (h) | – | 2.8 |
| $t\frac{1}{2}\beta$ (h) | – | 33 |
| $k_{10}$ ($min^{-1}$) | – | $5.5 \times 10^{-4}$ |
| $k_{12}$ ($min^{-1}$) | – | $1.3 \times 10^{-3}$ |
| $k_{21}$ ($min^{-1}$) | – | $2.6 \times 10^{-3}$ |

[a]Model-independent and dependent pharmacokinetic parameters were derived as described in Materials and methods.

Thus, the contribution of the $\alpha$-phase to total ICI D0490 AUC can be calculated as $A/\alpha$. The value so obtained, 3.9 mg $ml^{-1}$ $min^{-1}$, in comparison to the total AUC, 73 mg $ml^{-1}$ $min^{-1}$ (Table I), reveals at the $\alpha$-phase contributes only 5% of the total ICI D0490 AUC, another indication of the limited initial clearance of the immunotoxin. The calculated volume of the central compartment (63 ml $kg^{-1}$) is consistent with $V_1$ being the plasma volume whilst the volume of distribution of ICI D0490 at steady state (93 ml $kg^{-1}$) did not reach that of the calculated extracellular fluid volume, indicating little tissue penetration of the immunotoxin.

**Figure 2** Relationship between ICI D0490 dose and plasma levels 2 h **a** and 24 h **b** after administration. Each point is the mean of data from five mice. The lines are those given by linear regression analysis following logarithmic transformation of both the concentration and administered dose data.

**Table II** The effect of administration schedule on plasma levels of ICI D0490

| *Dose and schedule* | *Day* | *Time after ICI D0490 administration* | | |
|---|---|---|---|---|
| | | *2 h* | *24 h* | *48 h* |
| 1 mg kg$^{-1}$ day$^{-1}$ × 5 | 1 | 12.0 ± 2.1[a] | 6.3 ± 2.6 | ND[b] |
| | 3 | 10.4 ± 2.8 | 5.5 ± 1.8 | ND |
| | 5 | 7.8 ± 0.2 | 4.6 ± 0.9 | ND |
| 1.67 mg kg$^{-1}$ day$^{-1}$ × 3 | 1 | 13.9 ± 3.8 | 6.3 ± 3.4 | 2.4 ± 0.5 |
| | 3 | 12.6 ± 1.6 | ND | 2.5 ± 0.5 |
| | 5 | 11.8 ± 1.6 | 4.5 ± 0.8 | ND |
| 5 mg kg$^{-1}$ single | 1 | 12.7 ± 3.0 | 5.4 ± 2.2 | 1.9 ± 0.9 |

[a]Plasma concentration of ICI D0490 (μg ml$^{-1}$). to allow direct comparison plasma levels have been normalised to 1 mg kg$^{-1}$. ie: data for the × 3 and single dose schedules have been divided by 1.67 and 5, respectively. Data are the mean ± s.d. of observations from five mice.
[b]ND = Not determined.

*Effect of administered dose on the pharmacokinetics of ICI D0490 dosed iv*

To investigate the effect of dose on the pharmacokinetics of ICI D0490, plasma levels were measured at limited, representative, time points (2 and 24 h) following doses ranging from the $LD_{10}$ to 1/1000th $LD_{10}$. As shown in Figure 2, there was a good correlation between ICI D0490 dose and plasma concentrations at both time points and the slope of the relationships, when both concentration and dose data were logarithmically transformed, was unity (2 h = 0.99 ± 0.04; 24 h = 1.02 ± 0.07, mean ± SE). ICI D0490 could only be detected at 2 h following doses of 0.005 and 0.01 mg kg$^{-1}$. Hence the data obtained from this study indicate that the pharmacokinetics of ICI D0490 are linearly related to dose over at least a 100-fold range of doses from 0.05 mg kg$^{-1}$ to 5 mg kg$^{-1}$.

*Effect of repeated administration on the plasma levels of ICI D0490*

To investigate possible alterations in ICI D0490 clearance following repeated administration, a single iv bolus dose of 5 mg kg$^{-1}$ was compared to 1 mg kg$^{-1}$ day$^{-1}$ × 5 and 1.67 mg kg$^{-1}$ given on days 1, 3 and 5. As shown in Table II, no accumulation of ICI D0490 in the plasma over the period studied was seen at these dose levels. This is surprising since following a single dose of 2.5 mg kg$^{-1}$ the concentration of ICI D0490 in the plasma at 24 h (15 μg ml$^{-1}$) was 37% of the extrapolated concentration at time zero (Figure 1). Normalising the data to a dose of 1 mg kg$^{-1}$, this is equivalent to 5.6 μg ml$^{-1}$ which is within the observed range of ICI D0490 plasma levels 24 h after the first dose of 1 mg kg$^{-1}$ (6.3 ± 2.6 μg ml$^{-1}$, Table II). However, on repeated dosing at 1 mg kg$^{-1}$ no accumulation of ICI D0490 was observed. If anything, 2 h plasma levels appear to be lower following repeated administration of ICI D0490 (Table II). Taken together, the lack of ICI D0490 accumulation over 5 days and the apparent decrease in plasma levels at 2 h suggests that plasma clearance of the immunotoxin may be induced by repeated administration.

### Discussion

The experiments reported here show that ICI D0490 has favourable pharmacokinetic properties when administered intravenously to mice. Following a single iv dose of 2.5 mg ICI D0490 kg$^{-1}$ plasma levels could be fitted to a two compartment open model without distortion of the data. There was little initial clearance from the plasma and a relatively long α-phase half-life was observed. The volume of the central compartment approximates to that of the plasma volume in mice and hence during the initial 2 h period there is very little distribution of ICI D0490 from the plasma. In the light of earlier studies, the reduced initial clearance of ICI D0490 is probably a reflection of the aglycosyl nature of the recombinant ricin A-chain moiety of the immunotoxin (Thorpe *et al.*, 1988; Trown *et al.*, 1991; Wawrzynczak *et al.*, 1991*b*).

The second notable feature of the pharmacokinetics of ICI D0490 is the long elimination phase half-life of 33 h. To our knowledge, this is the longest β-phase half life so far reported for an immunotoxin in rodents and this property is presumably a reflection of the stable disulphide linker moiety of the ICI D0490. This is consistent with the findings of Thorpe and colleagues who demonstrated that, whilst the α-phase half life and plasma clearance were heavily influenced by A-chain glycosylation, the β-phase half life is a function of steric hindrance to cleavage of the disulphide bond (Thorpe *et al.*, 1988). Similar results were obtained by Worrell *et al.* (1986) and these latter authors developed the methyl-hindered linker used in the construction of ICI D0490. The potential importance of a long β-phase half life for immunotoxins has recently been underlined by modelling studies which suggest that half life can be an important as antibody binding affinity as a determinant of tumour localisation (Sung *et al.*, 1990). Furthermore, in their preclinical study, Thorpe *et al.* (1988) demonstrated that immunotoxins with a methyl-hindered linker had greater antitumour activity, when given as an equimolar dose, than immunotoxins with unhindered linkers.

In summary, for ICI D0490, both limited initial clearance and a prolonged β-phase half-life contribute to good plasma persistence and hence prolonged exposure of tumour cells to immunotoxin. Prolonged exposure of tumours should in turn aid penetration of ICI D0490 and hence maximise the likelihood of antitumour activity. However, it should be noted that the CA242 antigen is not expressed in mouse tissues and hence any influence of tissue CA242 levels on the pharmacokinetics of ICI D0490 would not have been detected in the experiments described here. Studies of the pharmacokinetics of ICI D0490 in nude mice bearing CA242 positive and negative human tumour xenografts are under way and preliminary results indicate no major differences.

Following a dose of 50% of the $LD_{10}$, the plasma levels of ICI D0490 were sustained at over 20 μg ml$^{-1}$ for 8 h and at over 1 μg ml$^{-1}$ for 96 h (Figure 1). This latter concentration is in excess of the level required *in vitro* to produce greater than 99.9% cell kill in a clonogenic assay using either CoLo201 or CoLo205 human colorectal tumour cells. Furthermore, 2.5 mg ICI D0490 kg$^{-1}$ is a dose that gives long growth delays (> 30 days) in a nude mouse xenograft model with the CoLo201 tumour that expresses the CA242 antigen (J. Calvete, unpublished results). However, no anti-tumour activity with this dose of ICI D0490 was observed against xenograft tumours not expressing CA242 antigen.

When a wide range of single doses of ICI D0490 was studied it was evident that there was a linear relationship between the dose given and plasma levels achieved, at 2 and 24 h, time points that reflect both phases of immunotoxin clearance. Thus, the pharmacokinetics of a low dose of ICI D0490 should allow the prediction of doses required to achieve target plasma levels. Dose escalation in early clinical trials, on the basis of plasma level data, has been widely advocated for cytotoxic drugs (Newell, 1990) although the role of this approach in studies with immunotoxins remains unclear. Given the limitations of preclinical toxicity models, initial doses of immunotoxins in Phase I trials should be conservative. However, once initial safety is established, dose escalation should be performed as quickly as possible. With an understanding of the immunotoxin concentrations required for *in vitro* cytotoxicy, and the plasma levels seen in mice following administration of therapeutic doses, it may be possible to guide dose escalation in patients. Whilst attractive, pharmacokinetically-guided dose escalation can only be attempted if a linear relationship exists between dose given and plasma level achieved. On the basis of the results presented in Figure 2, the pharmacokinetics of ICI D0490 would appear to be linearly related to dose and hence pharmacokinetics monitoring will be performed as part of the Phase I trial of the drug with a view to guiding dose escalation. An important aspect of pharmacokinetically-guided dosing is that pre-clinical and clinical studies should be comparable in terms of route and schedule. Initial phase I studies with ICI D0490 will involve single dose intravenous bolus administration.

In conclusion, these pharmacokinetic studies with ICI D0490 have shown it to be an extremely stable immunotoxin. Parallel pre-clinical studies, in the rat and a primate, support this conclusion (Dr J. Lynch, personal communication). Thus the clinical trial of ICI D0490, will represent an evaluation of possibly the most persistent immunotoxin yet studied for the therapy for cancer.

This work was supported in part by the North of England Cancer Research Campaign. The authors are grateful to Professor A.H. Calvert and Dr M.S. Rose for their continued enthusiasm and support. In addition, the authors wish to acknowledge the helpful advice given by Dr J. Lynch and Dr R. Smith.

### References

BAECKSTROM, D., HANSSON, G.C., NILSSON, O., JOHANSSON, C., GENDLER, S.J. & LINDHOLM, L. (1991). Purification and characterisation of a membrane-bound and a secreted mucin-type glycoprotein carrying the carcinoma-associated sialyl-Le epitope on distinct core proteins. *J. Biol. Chem.*, **266**, 21537–21547.

BALDWIN, R.W. & BYERS, V.S. (1987). Monoclonal antibody targeting of cytotoxic agents for cancer therapy. In *Immunology of Malignant Diseases*, Byers, V.S. (ed) pp 44–54. MTP Press: Lancaster, UK.

BLAKEY, D.C. & THORPE, P.E. (1986). Effect of chemical deglycosylation on the *in vivo* fate of ricin A-chain. *Cancer Drug Del.*, **3**, 189–196.

BLAKEY, D.C., WATSON, G.J., KNOWLES, P.P. & THORPE, P.E. (1987). Effect of chemical deglycosylation of ricin A-chain on the *in vivo* fate and cytotoxic activity of an immunotoxin composed of ricin A-chain and anti-Thy 1.1. antibody. *Cancer Res.*, **47**, 947–952.

BLAKEY, D.C., SKILLETER, D.N., PRICE, R.J., WATSON, G.J., HART, L.I., NEWELL, D.R. & THORPE, P.E. (1988). Comparison of the pharmacokinetics and hepatotoxic effects of saporin and ricin A-chain immunotoxins on murine liver parenchymal cells *Cancer Res.*, **48**, 7072–7078.

BOURRIE, B.J.P., CASELLAS, P., BLYTHMAN, H.E. & JANSEN, F.K. (1986). Study of the plasma clearance of antibody-ricin A-chain immunotoxins. *Eur. J. Biochem.*, **155**, 1–10.

BYERS, V.S., PIMM, M.V., PAWLUCZYK, I.Z.A., LEE, H.M., SCANNON, P.J. & BALDWIN, R.W. (1987). Biodistribution of ricin A-chain monoclonal antibody 791T/36 immunotoxin and influence of hepatic blocking agents. *Cancer Res.*, **47**, 5277–5283.

BYERS, V.S., RODVIEN, R., GRANT, K., DURRANT, L.G., HUDSON, K.H., BALDWIN, R.W. & SCANNON, P.J. (1989). Phase I study of monoclonal antibody-ricin A-chain immunotoxin XomaZyme-791 in patients with metastatic colon cancer. *Cancer Res.*, **49**, 6153–6160.

ENDO, Y., MITSUI, K., MOTIZUKI, M. & TSURUGI, K. (1987). The mechanism of action of ricin and related toxic lectins on eukaryotic ribosomes. *J. Biol. Chem.*, **262**, 5908–5912.

ENDO, Y. & TSURUGI, K. (1987). RNA N-glycosidase activity of ricin A-chain. *J. Biol. Chem.*, **262**, 8128–8130.

FITZGERALD, D. & PASTAN, I. (1989). Targeted toxin therapy for the treatment of cancer. *JNCI*, **81**, 1455–1463.

FUJIMORI, K., COVELL, D.G., FLETCHER, J.E. & WEINSTEIN, J.H. (1989). Modelling analysis for the global and microscopic distribution of immunoglobulin G, $F(ab')_2$ and Fab in tumours. *Cancer Res.*, **49**, 5656–5663.

GOULD, B.J., BOROWITZ, M.J., GROVES, E.S., CARTER, P.W., ANTHONY, D., WEINER, L.M. & FRANKEL, A.E. (1989). Phase I study of an anti-breast cancer immunotoxin by continuous infusion: report of a target toxic effect not predicted by animal studies. *JNCI*, **81**, 775–781.

HERTLER, A.A., SCHLOSSMAN, D.M., BOROWITZ, M.J., LAURENT, G., JANSEN, F.K., SCHMIDT, C. & FRANKEL, A.E. (1988). A Phase I study of T101-ricin A chain immunotoxin in refractory chronic lymphocytic leukaemia. *J. Biol. Resp. Mod.*, **7**, 97–113.

KUUSELA, P., HAGLAND, C. & ROBERTS, P.J. (1991). Comparison of a new tumour marker CA242 with CA19-9, CA50 and caricinoembryonic antigen (CEA) in digestive tract diseases. *Br. J. Cancer*, **63**, 636–640.

LARSON, L.N., JOHANSSON, C., LINDHOLM, L. & HOLMGREN, J. (1988). Mouse monoclonal antibodies for experimental immunotherapy promote killing of tumour cells. *Int. J. Cancer*, **42**, 877–882.

NEWELL, D.R. (1990). Phase I clinical studies with cytotoxic drugs: pharmacokinetic and pharmacodynamic considerations. *Br. J. Cancer*, **61**, 189–191.

PAI, L.H., BOOKMAN, M.A., OZOLS, R.F., YOUNG, R.C., SMITH, J.W., LONGO, D.L., GOULD, B., FRANKEL, A., MCCLAY, E.F., HOWELL, S., REED, E., WILLINGHAM, M.C. FITZGERALD, D.J. & PASTAN, I. (1991). Clinical evaluation of intraperitoneal *Pseudomonas* exotoxin immunoconjugate OVB3-PE in patients with ovarian Cancer. *J. Clin. Oncol.*, **9**, 2095–2103.

SPITLER, L.E. DEL RIO, M., KNENTIGAN, A., WEDEL, N.I., BROPHY, N.A., MILLER, L.L., HARKONEN, W.S., ROSENDORF, L.L., LEE, H.M., MISCHAK, R.P., KAWAHATA, R.T., STOUDEMIRE, J.B., FRADKIN, L.B., BAUTISTA, E.E. & SCANNON, P.J. (1987). Therapy of patients with malignant melanoma using a monoclonal antimelanoma antibody-ricin A-chain immunotoxin. *Cancer Res.*, **47**, 1717–1723.

SUNG, C., YOULE, R.J. & DEDRICK, R.L. (1990). Pharmacokinetic analysis of immunotoxin uptake in solid tumours: role of plasma kinetics, capillary permeability and binding. *Cancer Res.*, **50**, 7382–7392.

THORPE, P.E., WALLACE, P.M., KNOWLES, P.P., RELF, M.G., BROWN, N.F., WATSON, G.J., KNYBA, R.E., WAWARZYNCZAK, E.J. & BLAKEY, D.C. (1987). New coupling agents for the synthesis of immunotoxins containing a hindered disulphide bond with improved stability *in vivo*. *Cancer Res.*, **47**, 5924–5931.

THORPE, P.E., WALLACE, P.M., KNOWLES, P.P., RELF, M.G., BROWN, A.N.F., WATSON, G.J., BLAKEY, D.C. & NEWELL, D.R. (1988). Improved antitumour effects of immunotoxins prepared with deglycosylated ricin A-chain and hindered disulphide linkages. *Cancer Res.*, **48**, 6396–6403.

TROWN, P.W., REARDAN, D.T., CARROLL, S.F., STOUDEMIRE, J.B. & KAWAHATA, R.T. (1991). Improved pharmacokinetics and tumour localization of immunotoxins constructed with the $M_r$ 30,000 form of ricin A chain. *Cancer Res.*, **41**, 4219–4225.

VITETTA, E.S., STONE, M., AMLOT, P., FAY, J., MAY, R., TILL, M., NEWMAN, J., CLARK, P., COLLINS, R., CUNNINGHAM, D., GHETIE, V., UHR, J.W. & THORPE, P.E. (1991). Phase I immunotoxin trial in patients with B-cell lymphoma. *Cancer Res.*, **51**, 4052–4058.

VITTETA, E.S. & THORPE, P.E. (1991). Immunotoxins. In *Biologic Therapy of Cancer*, DeVita, V.T. (ed) pp. 483–495. J.B. Lippincott Company: Philidelphia.

WAWRZYNCZAK, E.J. (1991). Systemic immunotoxin therapy of cancer: advances and prospects. *Br. J. Cancer*, **64**, 624–630.

WAWRZYNCZAK, E.J., CUMBER, A.J., HENRY, R.V., MAY, J., NEWELL, D.R., PARNELL, G.D., WORRELL, N.R. & FORRESTER, J.A. (1990). Pharmacokinetics in the rat of a panel of immunotoxins made with abrin A-chain, ricin A-chain, gelonin and momordin. *Cancer Res.*, **50**, 7519–7526.

WAWRZYNCZAK, E.J., HENRY, R.V., CUMBER, A.J., PARNELL, G.D., DERBYSHIRE, E.J. & ULBRICH, N. (1991*a*). Biochemical, cytotoxic and pharmacokinetic properties of an immunotoxin composed of a mouse monoclonal antibody Fib75 and the ribosome-inactivating protein α-sarcin from *Aspergillus giganteus*. *Eur. J. Biochem.*, **196**, 203–209.

WAWRZYNCZAK, E.J., CUMBER, A.J., HENRY, R.V. & PARNELL, G.D. (1991*b*). Comparative biochemical, cytotoxic and pharmacokinetic properties of immunotoxins made with native ricin A-chain, ricin-$A_1$ chain and recombinant ricin A-chain. *Int. J. Cancer*, **47**, 130–135.

WELLING, P.G. (1986). Pharmacokinetics, processes and mathematics. ACS Monograph 185, Washington, DC.

WEINER, L.M., O'DWYER, J., KITSON, J., COMIS, R.I., FRANKEL, A.E., BAUER, R.J., KONRAD, M.S. & GROVES, E. (1989). Phase I evaluation of an anti-breast carcinoma monoclonal antibody 260F9-recombinant ricin A-chain immunotoxin. *Cancer Res.*, **49**, 4062–4067.

WORRELL, N.R., CUMBER, A.J., PARNELL, G.D., MIRZA, A., FORRESTER, J.A. & ROSS, W.C.J. (1986). Effect of linkage variation on pharmacokinetics of ricin A-chain-antibody conjugates in normal rats. *Anti-Cancer Drug Design*, **1**, 179–188.

WRIGHT, A.F., BLAKEY, D.C., FITTON, J.E., GREEN, T.P., HALL, S.M., LYNCH, J. VALCACCIA, B.E. & ROSE, M.S. (1992). *Br. J. Cancer*, **65**, (suppl XVI) P145.